# Deletion variant near *ZNF389* is associated with control of ovine lentivirus in multiple sheep flocks

**DOI:** 10.1111/age.12107

**Published:** 2013-12-05

**Authors:** S N White, M R Mousel, J O Reynolds, L M Herrmann-Hoesing, D P Knowles

**Affiliations:** * Animal Disease Research Unit, Agricultural Research Service, U.S. Department of AgriculturePullman, WA, 99164, USA; † Department of Veterinary Microbiology and Pathology, Washington State UniversityPullman, WA, 99164, USA; † U.S. Sheep Experiment StationDubois, ID, 83423, USA

**Keywords:** host control, maedi-visna virus, marker-assisted selection, ovine lentivirus, ovine progressive pneumonia, susceptibility, *ZNF165*, *ZNF192*, *ZNF389*, *ZSCAN16*

## Abstract

Ovine lentivirus (OvLV) is a macrophage-tropic lentivirus found in many countries that causes interstitial pneumonia, mastitis, arthritis and cachexia in sheep. There is no preventive vaccine and no cure, but breed differences suggest marker-assisted selective breeding might improve odds of infection and control of OvLV post-infection. Although variants in *TMEM154* have consistent association with odds of infection, no variant in any gene has been associated with host control of OvLV post-infection in multiple animal sets. Proviral concentration is a live-animal diagnostic measure of OvLV control post-infection related to severity of OvLV-induced lesions. A recent genome-wide association study identified a region including four zinc finger genes associated with proviral concentration in one Rambouillet flock. To refine this region, we tested additional variants and identified a small insertion/deletion variant near *ZNF389* that showed consistent association with proviral concentration in three animal sets (*P *<* *0.05). These animal sets contained Rambouillet, Polypay and crossbred sheep from multiple locations and management conditions. Strikingly, one flock had exceptionally high prevalence (>87%, including yearlings) and mean proviral concentration (>950 copies/μg), possibly due to needle sharing. The best estimate of proviral concentration by genotype, obtained from all 1310 OvLV-positive animals tested, showed insertion homozygotes had less than half the proviral concentration of other genotypes (*P *<* *0.0001). Future work will test additional breeds, management conditions and viral subtypes, and identify functional properties of the haplotype this deletion variant tracks. To our knowledge, this is the first genetic variant consistently associated with host control of OvLV post-infection in multiple sheep flocks.

Ovine lentivirus (OvLV), also known as ovine progressive pneumonia or maedi-visna virus, infects approximately one-quarter of U.S. sheep and half of all range flocks contain OvLV-positive individuals (InfoSheet 2003). The virus is widespread in sheep from many countries around the world (Thormar 2005; Leroux *et al*. 2010; Blacklaws 2012). Infected sheep can have varying degrees of the following symptoms: dyspnea, mastitis, cachexia, arthritis and/or encephalitis (Leroux *et al*. 2010; Blacklaws 2012). Production losses stem from lamb mortality (Arsenault *et al*. 2003), lower lamb weights from older infected ewes (Keen *et al*. 1997; Arsenault *et al*. 2003), early culling (Peterhans *et al*. 2004) and export restrictions (Reina *et al*. 2009). Current intervention strategies include separation of lambs from dams at birth to prevent transmission and test/cull methods (Houwers *et al*. 1983, 1984). However, these methods are not cost-effective (Houwers 1990), and a vaccine preventing infection has not been developed (Reina *et al*. 2009).

Breed differences in both odds of infection and control of virus once infected have suggested a host genetic basis for susceptibility to OvLV (Herrmann-Hoesing *et al*. 2008; Blacklaws 2012), and recent work has identified the first genetic marker test for odds of OvLV infection based on the *TMEM154* gene (Heaton *et al*. 2012, 2013). However, no genetic marker test has been validated for control of OvLV once infected. A genome-wide association study (GWAS) identified several genomic regions associated with proviral concentration (White *et al*. 2012), a live-animal diagnostic measure of proviral replication that is predictive of pathological lesion severity (Herrmann-Hoesing *et al*. 2009). We aimed to identify one or more genetic markers within one genomic region from the GWAS that would have consistent association in multiple additional groups of sheep.

Proviral concentrations were measured by a validated assay (Herrmann-Hoesing *et al*. 2007) in animal sets totaling 2170 ewes (no males); each animal set was chosen for high OvLV levels (Table1). These included purebred Rambouillet, Polypay and Columbia sheep from Idaho sampled in 2004 and 2008, and purebred Polypay sheep from Iowa sampled in 2009 described previously (Herrmann-Hoesing *et al*. 2008; Heaton *et al*. 2012; White *et al*. 2012). The generation interval in the Idaho populations was approximately 2–3 years, and domestic sheep from Idaho in 2004 and 2008 did not include any animals shared between sample groups. Additionally, privately owned commercial Rambouillet–Columbia crossbred sheep from Montana were sampled in 2009 (Table1). In this animal set, more than 90% of animals over the age of 1 year and 95% of animals over the age of 2 years were OvLV positive. Animal care and handling procedures for research animals were approved by Washington State University Institutional Animal Care and Use Committee (Permit 3171) and/or by U.S. Sheep Experiment Station Care and Use Committee (Permits 10-06 and 10-07).

**Table 1:** Animal sets used for association analysis

Animal set	Breed(s)	Age range (years)	Location	Collection date	Total number	Number OvLV positive^1^	OvLV prevalence (%)	Mean proviral concentration^2^ among OvLV positives
1	Rambouillet	1–5	Idaho	2008	372	157	42.2	52.9
2	Polypay	1–5	Idaho	2008	401	158	39.4	169.3
3	Columbia	1–5	Idaho	2008	134	62	46.3	225.1
4	Rambouillet, Polypay, Columbia	3–6	Idaho	2004	331	208	62.8	229.2
5	Polypay	1–8	Iowa	2009	321	192	59.8	221.9
6	Crossbred Rambouillet–Columbia	1–8	Montana	2009	611	533	87.2	971.4

1 OvLV positive as defined by positive proviral concentration.

2 Means were calculated on log_10_-transformed proviral concentrations to reduce influence of the highest proviral concentrations (outliers) and reverse-transformed to viral copies/μg DNA scale.

Multiple haplotype-tagging genetic variants were identified from resequencing of sheep HapMap animals (Kijas *et al*. 2012) provided under the Toronto guidelines for data users pre-publication (Birney *et al*. 2009). Genotyping variants from a four gene region near 29.5 Mb on ovine chromosome 20 including *ZNF165*, *ZSCAN16*, *ZNF192* and *ZNF389* (Archibald *et al*. 2010) were performed using TaqMan assays (Life Technologies) according to the manufacturer's specifications (Table S1). A *ZNF389* deletion (*NC_019477.1*:g.29500068_29500069delAT ovine chromosome 20, NCBI dbSNP ss748775100, hereafter referred to as *ZNF389* deletion variant or deletion variant) was genotyped using a TaqMan assay with CGAATGGATCTTCAAGGCTTA and CAGCTTTTCCATGCAGAGTC as amplification primers, TCCAATAAAATATGAC as a probe labeled with VIC dye and TCCAATAAAATGACTT as a probe labeled with FAM dye (Table S1). Details on genotyping assays for other variants may be found in Table S1. When significant association was observed in animal set 1, containing Rambouillets from the original GWAS (White *et al*. 2012), genotyping was performed on additional animal sets. Figure S1 shows genomic positions for all markers listed in Table S1. Observed insertion allele frequencies by animal set were 55.1% (in animal set 1), 49.3%, 41.0%, 41.8%, 25.4% and 43.5% respectively. Table S2 shows genotype counts for the *ZNF389* deletion in each animal set.

Statistical analysis employed the general linear models procedure of sas 9.2 (SAS Institute) with log_10_-transformed proviral concentrations as the dependent variable as previously described (Herrmann-Hoesing *et al*. 2008). Independent predictors included genotype, breed and age in years, which was treated as categorical to account for nonlinearity in proviral concentration at advanced age. Similar logistic regression models were used in the logistic procedure of sas 9.2 to analyze infection status as the dependent variable. An omnibus analysis to estimate effect size was performed using the mixed procedure of sas 9.2 to analyze log_10_-transformed proviral concentration as the dependent variable, fixed categorical effects of genotype, breed, age in years and a random effect of animal set. A *P*-value <0.05 was considered significant for all the analyses.

The most significant haplotype in animal set 1 included s65956 from OvineSNP50 (Kijas *et al*. 2012) as well as markers in or near *ZNF389*. A two base-pair *ZNF389* deletion variant in the 5′ genomic region of *ZNF389* was strongly associated with proviral concentration in animal set 1 (*P *=* *0.0001; Table2). Notably, the *ZNF389* deletion variant accounted for the entire effect size of the GWAS marker s65956 in this animal set. Although s65956 was not associated with proviral concentration in animal set 2 (*P *>* *0.52) in a previous GWAS (White *et al*. 2012), the *ZNF389* deletion variant was associated with proviral concentration in all the animal sets with more than 35 OvLV-positive insertion homozygotes (Table2; Fig. S1). However, the deletion variant was not associated with odds of OvLV infection (all *P *>* *0.05 for estimable animal sets with more than 35 OvLV-positive insertion homozygotes; see Table S3). Interestingly, the insertion allele frequency was higher in Rambouillets than in other breeds (Table S2), and this generally matches the trend of breed differences in OvLV proviral concentration (Herrmann-Hoesing *et al*. 2008).

**Table 2:** Association of *ZNF389* deletion variant g.29500068_29500069delAT with proviral concentration by animal set

Animal set	Adjusted mean proviral concentration^1^ by genotype			*P*-value
	II^2^	ID^2^	DD^2^	
1	22.4	46.6	175.9	0.0001
2	54.5	235.4	133.2	0.012
3	–	–	–	NS^3^
4	–	–	–	NS^3^
5	–	–	–	NS^3^
6	632.4	1388.0	1460.5	0.0009
All	123.3	263.1	265.0	<0.0001

1 Adjusted means were derived from models accounting for animal age (and breed, if multiple breeds present in the animal set) and were reverse-transformed to viral copies/μg DNA scale.

2 II, insertion homozygote; ID, insertion/deletion heterozygote; DD, deletion homozygote.

3 Not significant (*P *>* *0.05) with less than 35 OvLV-positive II homozygotes.

Despite differences in location, breeds and management (Table1), consistent association was observed between the *ZNF389* deletion variant and proviral concentration in some of the most common breeds and breed types present on the U.S. range (Table2). For example, in animal sets 1–3, needle sharing during vaccination was carefully avoided to reduce iatrogenic transmission. However, in animal set 6, needle sharing was practiced during vaccination, and observed proviral concentration was much higher (Table1). Further, the influence of other genes can be a concern. In animal set 6, the degree of Rambouillet influence among crossbred sheep was variable but not documented. As breed differences in OvLV proviral concentration have been observed between Columbia and Rambouillet sheep (Herrmann-Hoesing *et al*. 2008), the unknown fraction of Rambouillet inheritance might obscure differences due to other genes and reduce the estimate of effect size. Nonetheless, the best estimate of effect size based on all 1310 OvLV-positive animals showed insertion homozygotes had less than half the adjusted mean proviral concentration compared with other genotypes (Table2).

Currently available genetic tests based on *TMEM154* address odds of OvLV infection (Heaton *et al*. 2012, 2013) and have been associated with proviral concentration in one sheep flock (F. Alshanbari and S. White, unpublished data). However, no variant has demonstrated consistent association with proviral concentration in multiple animal sets. The current results suggest the deletion variant might have predictive value for OvLV proviral concentration under a range of conditions including breeds, locations and virus strains similar to those examined here (Dekkers 2004). The only animal set with the insertion as the major allele was animal set 1, composed of Rambouillets. All other animal sets had high minor allele frequencies (25–49%), especially those with high degrees of Rambouillet influence such as the Rambouillet–Columbia crossbred ewes in animal set 6 (43.5%; see also Table S2). This suggests genotype frequencies of insertion homozygotes could be changed quickly under selection for rapid genetic progress.

The functional importance of this genomic region for control of OvLV is currently unknown. Zinc finger proteins have characteristic nucleic acid-binding domains and often serve to regulate gene transcription. One or more of the zinc finger genes in this genomic region might act through transcriptional regulation of host genes that could restrict proviral replication of OvLV, such as TRIM5*α* (Jauregui *et al*. 2012). Because zinc finger genes can directly restrict lentiviruses (Zhu *et al*. 2011) and over evolutionary time they can diversify to address retroviral challenges (Thomas & Schneider 2011), it also possible that one or more of the genes in this region restrict proviral replication more directly.

One hypothesis is that genetic selection for reduced OvLV proviral concentration might complicate efforts to detect and eliminate OvLV. However, it is not known whether there is a relationship between transmission and proviral concentration among OvLV-positive animals. Proviral concentration is a measure of proviral replication, and if low proviral concentration is related to low viral transmission, then interventions to reduce proviral concentration could assist in reducing viral transmission. Further, the known correlation between proviral concentration and lesion severity (Herrmann-Hoesing *et al*. 2009) suggests that animals selected for reduced proviral concentration may also have reduced disease progression and severity, and this would be of value to mitigate many commercial losses due to OvLV.

To our knowledge, this is the first report of a genetic variant consistently associated with control of OvLV post-infection in multiple sheep flocks. These results implicate one or more functional variants nearby on the same haplotype that play a biological role related to OvLV proviral concentration. Additional work will be required to identify mechanism(s) and to determine whether better markers exist in the same region.

## Supporting Information

Figure S1 Gene and marker placement including *ZNF389* deletion variant g.29500068_29500069delAT. Markers are indicated by vertical white bars and are shown in the same order as Table S1.

Table S1 Genetic markers tested and genotyping reagents.

Table S2 *ZNF389* deletion variant g.29500068_29500069delAT genotype counts by animal set.

Table S3 Association of *ZNF389* deletion variant g.29500068_29500069delAT with proviral concentration for animal sets 3, 4 and 5.
