# Supplementary material for: Deletion variant near ZNF389 is associated with control of ovine lentivirus in multiple sheep flocks
Source: Anim Genet. 2013 Dec 5;45(2):297–300. doi: 10.1111/age.12107 (PMC4225466; doi:10.1111/age.12107)

Figure S1. Gene and marker placement including *ZNF389* deletion variant g.29500068_29500069delAT. Markers are indicated by vertical white bars and are shown in the same order as Table S1.

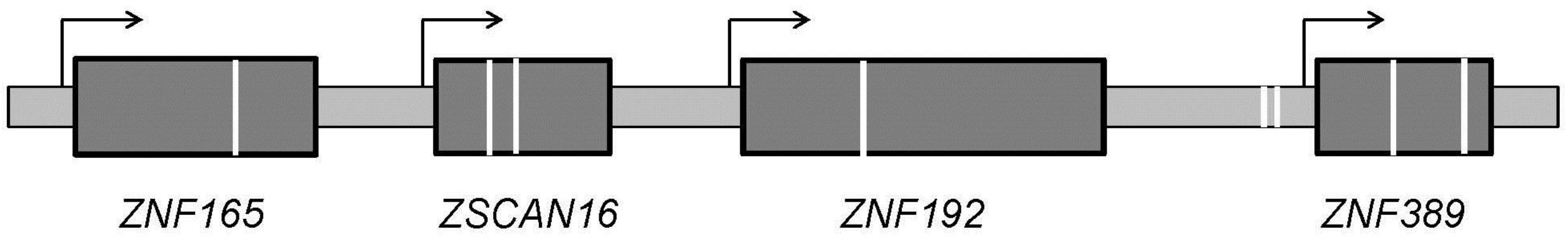

Supplement: Figure S1 — Gene and marker placement including ZNF389 deletion variant g.29500068_29500069delAT. Markers are indicated by vertical white bars and are shown in the same order as Table S1. [file age0045-0297-sd1.pdf]
