# Supplementary material for: Deletion variant near ZNF389 is associated with control of ovine lentivirus in multiple sheep flocks
Source: Anim Genet. 2013 Dec 5;45(2):297–300. doi: 10.1111/age.12107 (PMC4225466; doi:10.1111/age.12107)
Supplement: Table S1 — Genetic markers tested and genotyping reagents. [file age0045-0297-sd2.pdf]

Table S1. Genetic markers and genotyping reagents.

Sequence variant[1,2]	dbSNP identifiers	Annotation[3]	Amplification Primer 1	Amplification Primer 2	Probe 1[4]	Probe 2[5]
g.29565141G>A	ss748775096	*ZNF165* A315V	GTGACCAAAGTTTCAAATGGAATTCAGA	AGGTTTGGGTTCTTGAGAGATTTCC	TTCTCCAGCATAAATT	TTTCTCCAACATAAATT
g.29542468G>A	ss748775097	*ZSCAN16* R127Q	TGGAGGATCTGGAGAGAAAGCTT	CTGAAAACTCACAGCCAAATGATCA	ATGAACCTCAAAAGCA	ATGAACCTCGAAAGCA
g.29540906T>C	ss748775098	*ZSCAN16* I156M	CCTTGAGAAGGCCACATGAGT	GCTCCTGCCAGGTCTTCTTG	CATTGACTATGCAGCTTC	CATTGACTATCCAGCTTC
g.29551003T>C	ss748775099	*ZNF192* near splice site Exon 4	CCAGTGTAATTACTGTGGGAAAGCT	ACTGGTAAGGTTTCTCTCCTGTGT	TGTCGAATAAGGCCTGAGTT	TCGAATAAGGCCCGAGTT
g.29500068_29500069delAT*	ss748775100	*5' of ZNF389**	CGAATGGATCTTCAAGGCTTA	CAGCTTTTCCATGCAGAGTC	TCCAATAAAATATGAC	TCCAATAAAATGACTT
g.29499988G>A	ss748775101	*5' of ZNF389*	CTGTTTAGTCCCTACTCCTTTCACTTG	CTTTCTGAGACCTAAGCCTTGAAGA	TCCATTCGCTGGTTCC	CATTCGCCGGTTCC
g.29496098T>C	ss748775102	*ZNF389* N125S	GTGAGGGCACACCTGATTATACAT	CACACTAAATGCTTTGCCACATTCA	CAGAGAATCCACAATGGAGA	CAGAGAATCCACAGTGGAGA
g.29495990T>C	ss748775103	*ZNF389* G245D	ACCAGAGAATCCATACTGGAGAGAA	AGCTCCCACGGAATGCTTT	CCACACTCATCACACTC	CACACTCACCACACTC

[1]Nucleotide positions in reference to GenBank accession NC_019477.1.

[2]Order numbers (Applied Biosystems, Inc.) for reagents described: AHRSGUL, AHN1LZO, AHPAJ5W, AHUAC61, AHN1R6J, AHLJQC5, AHVJBC9, AHWR9JH

[3]Coding positions in reference to accessions NM_001191376.1 (*ZNF165*), NM_001034411.1 (*ZSCAN16*), NM_001100344.1 (*ZNF192*), and NM_001145131.1 (*ZNF389*).

[4]Probe labeled with VIC dye.

[5]Probe labeled with FAM dye.

*Deletion identified as consistently associated with OvLV proviral concentration
