# Supplementary material for: Deletion variant near ZNF389 is associated with control of ovine lentivirus in multiple sheep flocks
Source: Anim Genet. 2013 Dec 5;45(2):297–300. doi: 10.1111/age.12107 (PMC4225466; doi:10.1111/age.12107)
Supplement: Table S2 — ZNF389 deletion variant g.29500068_29500069delAT genotype counts by animal set. [file age0045-0297-sd3.pdf]

Table S2. *ZNF389* deletion variant g.29500068_29500069delAT genotype counts by animal set.

Animal Set	1	2	3	4	5	6	Total All Sets
Total II	104	63	16	50	15	109	357
Total ID	202	269	78	177	133	314	1173
Total DD	66	69	40	104	173	188	640
Overall Sum	372	401	134	331	321	611	2170
OvLV+ II	46	19	8	34	13	97	217
OvLV+ ID	85	107	36	117	86	279	710
OvLV+ DD	26	32	18	57	93	157	383
OvLV+ Sum	157	158	62	208	192	533	1310
