# Supplementary material for: Deletion variant near ZNF389 is associated with control of ovine lentivirus in multiple sheep flocks
Source: Anim Genet. 2013 Dec 5;45(2):297–300. doi: 10.1111/age.12107 (PMC4225466; doi:10.1111/age.12107)
Supplement: Table S3 — Association of ZNF389 deletion variant g.29500068_29500069delAT with proviral concentration for animal sets 3, 4 and 5. [file age0045-0297-sd4.pdf]

Table S3. Association of *ZNF389* deletion variant g.29500068_29500069delAT with proviral concentration for animal sets 3*, 4*, and 5*.

	Adjusted Mean Proviral Concentration[1] by Genotype			
Animal Set	II[2]	ID[2]	DD[2]	P-value
3	183.9	307.4	131.9	NS[3]
4	192.0	229.4	284.3	NS[3]
5	109.0	204.5	185.7	NS[3]

[1]Adjusted means were derived from models accounting for animal age (and breed, if multiple breeds present in the animal set), and were reverse-transformed to viral copies/µg DNA scale.
[2]II, insertion homozygote. ID, insertion/deletion heterozygote. DD, deletion homozygote.
[3]Not significant ($P>0.05$) with less than 35 OvLV-positive II homozygotes
*Note: Many of these means were derived from small numbers of individuals (as low as 8 ewes; see Table S2). Thus, means are expected to include some unstable estimates due to small sample size. Trends in these data, while present, are not expected to be as clear in animal sets with such sample size limitations.
